# Chemical Strategies to Custom-Modify α(1→3)-Fucosylated Glycan Motifs of the Human Glycocalyx

**DOI:** 10.1021/jacsau.5c00716

**Published:** 2025-10-06

**Authors:** Pedro Miguel Ascenso Vieira, Giacomo Biagiotti, Robert Sackstein, Barbara Richichi

**Affiliations:** † Department of Chemistry “Ugo Schiff”, University of Firenze, Via della Lastruccia 3-13, 50019 Sesto Fiorentino, (Florence), Italy; ‡ Department of Translational Medicine, and Translational Glycobiology Institute, Herbert Wertheim College of Medicine, Florida International University, Miami, Florida 33199, United States

**Keywords:** translational glycobiology, fucosyltransferases, glycan motifs, sialylated Lewis X, glycocalyx, glycomimetics, inhibitors, E-selectin ligand

## Abstract

In mammals, every cell is covered by a sugar coat called the “glycocalyx”, a meshwork created by sugar modifications of cell surface proteins and lipids. Essentially all cell membrane proteins and lipids contain oligosaccharide clusters known as “glycan motifs” that confer distinct functional properties on these respective glycoproteins or glycolipids. These motifs are generated by glycosyltransferases that assemble the component monosaccharides in a stereospecific and regiospecific fashion. Glycocalyx motifs bearing l-fucose in α(1→3) linkage to *N*-acetyl-glucosamine are found on a highly restricted subset of membrane glycoproteins and glycolipids, and changes in α(1→3)-fucosylation levels impact a wide range of physiologic and pathologic processes. Within this biological framework, we herein review the pivotal role of α(1→3)-fucosylation in cell biology and then comprehensively review the evolving chemical strategies to custom-modify α(1→3)-fucosylation of the glycocalyx to achieve highly specific control of human cell surface fucosylated glycan motifs. These efforts serve as a prime example of how the fine control of cell surface fucosylation can enable the generation of glycan-based precision therapeutics, driving forward the field of “translational glycobiology”.

## Graphical abstract

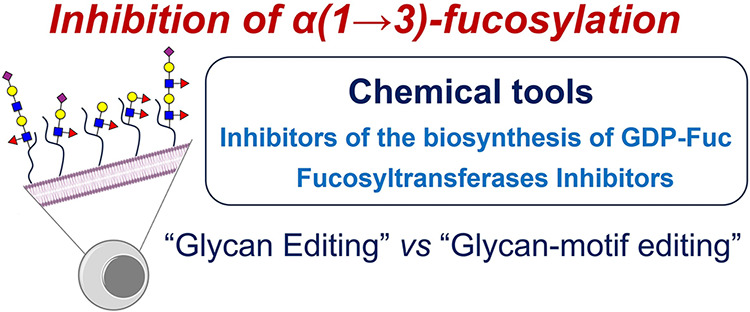

## Introduction

Glycosylation is a post-translational modification essential for both physiologic and pathologic cellular functions.^1^ Essentially all membrane proteins and lipids are heavily glycosylated, consisting of glycoproteins and glycolipids, respectively. This post-translational process is initiated within the endoplasmic reticulum (ER) and then continues into the Golgi apparatus, which is the predominant site of protein and lipid glycosylation within a cell.^2–5^ Unlike nucleic acid and protein biosynthesis, this process is not template-driven but is instead controlled by the elaboration of distinct enzymes (known as “glycosyltransferases” (GTs)) and results in the creation of glycans, *i.e*., complex carbohydrates. Glycosylation proceeds via a stepwise covalent/glycosidic attachment of pertinent monosaccharides (from the requisite “activated” donor nucleotide/lipid-derivative) to specific acceptor structures (*i.e*., carbohydrates, proteins, lipids, small RNA) recognized by specific GTs.^6–8^ Glycan structures are also modified by selective removal of monosaccharides by the action of other specialized enzymes named glycosidases (“glycan trimming”).^9^ Thus, the elaboration of a distinct glycan motif on a given cell surface reflects a balance between its biosynthesis and any (subsequent) glycosidase-mediated catabolic event(s).

On protein scaffolds, glycans are covalently attached to a nitrogen within asparagine (*i.e*., “*N*-glycans”)^10^ or to oxygen within serine and/or threonine residues (*i.e*., “*O*-glycans”),^11^ whereas glycans are linked solely via oxygen within lipid scaffolds.^12^ Less common *O*-glycosylation sites at the tyrosine and hydroxylysine residues have been documented in specific biological contexts.^13,14^ Then, more recently, structures named glycoRNA^15^ that consist of saccharides enriched with terminal monosaccharides such as sialic acid and fucose covalently linked to small RNA species have been identified in various cell types and mammalian species. Such glycosylated structures are still under investigation to further elucidate their assembly, structure, and function.

Most of the GTs, named “Leloir GTs”, use activated sugar donors in the form of mono- or diphosphate nucleotide-linked monosaccharides. Monosaccharides and nucleotides are matched with high specificity, and their biosynthesis is strictly controlled by multistep biosynthetic pathways that are regulated by both the monosaccharide metabolism and the availability of the activated donor nucleotide monosaccharides from both cellular and extracellular pools.^16^ Assembled donors are then transported into the Golgi or the ER through specific nucleotide transporters that are located only within the membrane of the organelle, where the pertinent GT is located. Once they enter the lumen of the Golgi/ER, the activated donors become accessible to the catalytic domain of the GTs.

Accordingly, the steps required to build glycans embedded within the glycocalyx are orchestrated by the convergence of multiple factors, including the availability of substrates and the spatial-temporal convergent localization of acceptor/glycosyltransferase/donor nucleotide monosaccharides within the Golgi/ER, along with cell signaling, gene transcription, and enzyme activity.

One important family of cellular GTs is the fucosyltransferases (FTs), of which 13 members have been described in humans.^17–21^ These enzymes are responsible for the installation of a l-fucose (Fuc) residue from a nucleotide-activated form of Fuc (donor substrate), *i.e*., guanosine diphosphate-fucose (GDP-fucose, GDP-Fuc), via α(1→2)-linkage to a d-galactose (Gal),^22^ via α­(1→3/4)- or α(1→6)-linkages to an *N*-acetyl-d-glucosamine (GlcNAc) on a large array of glycans, or *via O*-fucosylation to serine/threonine residues in epidermal growth factor (EGF)-like, thrombospondin type-1 repeat domains,^17,22^ and within the elastin microfibril interface domain of Multimerin-1.^23^

Within the family of FTs, this Perspective article will focus on the pivotal role of α(1→3)-FTs^17,19,21,24^ in the biosynthesis of diverse α(1→3)-fucosylated (sialyl)­lactosaminyl glycans known as “Lewis antigens”. This post-translational modification influences how cells interact with their environment, regulating various intercellular and intracellular biological processes, including signal transduction, cell–cell and cell–matrix adhesive interactions, intercellular communication, cell development, embryogenesis, and immune responses.^20,25–28^ Moreover, aberrant expression of α(1→3)-FTs also has significant implications for carcinogenesis and cancer metastasis.^29–32^ As such, aberrant cell surface α(1→3)-fucosylation serves as a biomarker for diagnosis and prognosis of malignancies, for tracking tumor load and response to therapy, and for the development of therapeutic interventions. For details about the involvement of specific α(1→3) fucosylated glycan motifs in disease conditions, we refer to more specific articles on this topic.^25,29–32^

In this context, we have recently highlighted the crucial difference between the biochemical modification(s) that grossly alter multiple types of glycan motifs embedded within the glycocalyx (the “glycan-editing approach”) *vs* the express intention to selectively regulate distinct cell surface glycans motifs (the “glycan-motif editing approach”).^33^ Here, we provide a roadmap for future investigations to enable the precision editing of cell surface α(1→3)-fucosylation using small molecules with the capacity to inhibit α(1→3)-FTs. Enormous efforts have been put forth to identify and design this class of compounds. However, there are still many obstacles that significantly impair drug discovery and development in this field.

Accordingly, we will provide a comprehensive overview of the state of the art, focusing on the synthetic development of small molecules for the inhibition of α(1→3)-fucosylation. Our overarching effort is to advance the application of glycan-based precision therapeutics in clinical medicine, a prime example of the colossal potential of the discipline known as “translational glycobiology”.

## Classification of the α(1→3)-Fucosyltransferases and Structure of the Related Glycan Motifs

α­(1→3)-FTs are Golgi-resident GTs and belong to the GT10 glycosyltransferase family of Carbohydrate-Active enZYmes (CAZy, https://www.cazy.org/). They are responsible for installing terminal l-fucose residues on neutral (unsialylated) “Type-2” acceptor as Gal-β(1→4)-GlcNAc-β-1-R (LacNAc) and on sialylated “Type-2” acceptor as Neu5Ac-α(2→3)-Gal-β(1→4)-GlcNAc-β-1-R (sLacNAc) (Figure 1).^21,24^

**Figure 1:**
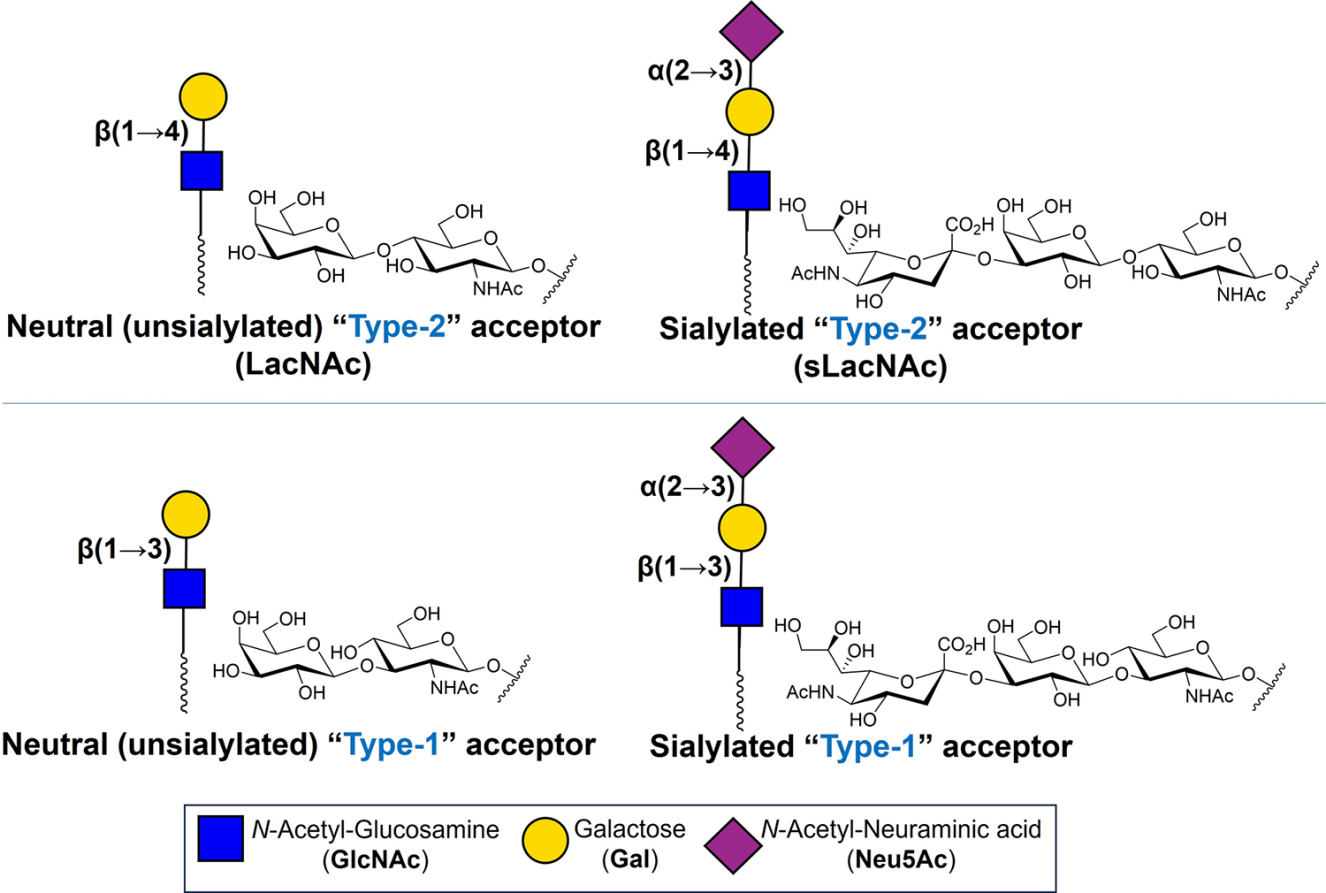
Schematic representation of the neutral (unsialylated) “Type-2” acceptor Gal-β(1→4)-GlcNAc-β-1-R (**LacNAc**) and sialylated “Type-2” acceptor Neu5Ac-α(2→3)-Gal-β(1→4)-GlcNAc-β-1-R (**sLacNAc**) lactosamines; these structures are the precursors of Lewis “X”-type lactosaminyl glycans that are created by the action of α(1→3)-FTs. Also depicted here are the isomeric neutral (unsialylated) “Type-1” acceptor Gal-β(1→3)-GlcNAc-β-1-R and sialylated “Type-1” acceptor Neu5Ac-α(2→3)-Gal-β(1→3)-GlcNAc-β-1-R lactosamines; these structures are the precursors of Lewis “A”-type lactosaminyl glycans created by the action of α(1→4)-FTs.

There are six human FTs that can catalyze the formation of an α(1→3)-linkage of a Fuc residue to a GlcNAc residue within a terminal lactosamine unit, and these are known as FTIII, FTIV, FTV, FTVI, FTVII, and FTIX. These enzymes shape the intracellular biosynthesis of relevant fucosylated glycan determinants, with each enzyme exhibiting specificity for distinct acceptor substrates (“Type-2” LacNAc or sLacNAc, Figure 1). Knowledge of their specific catalytic activity and their pattern(s) of glycan-motif creation is crucial for the evaluation of the biological impact of glycan-editing *vs* the glycan-motif editing effect mediated by synthetic inhibitors.

In particular, only human FTIII, FTV, FTVI, and FTVII can efficiently perform α(1→3)-fucosylation of sLacNAc to create the fucosylated sialyl-lactosaminyl glycan determinant known as “sialylated Lewis X” (sLe^X^, CD15s; Neu5Ac-α(2→3)-Gal-β(1→4)-[Fuc-α(1→3)]-GlcNAc-β-1-R, Table 1, entry c). The sLe^X^ determinant is an operationally critical glycocalyx motif in the regulation of leukocyte trafficking and in cancer metastasis because it is the prototypical binding determinant for the selectin family of adhesion molecules (comprised of E-selectin (CD62E), L-selectin (CD62L), and P-selectin (CD62P)).^33^ Importantly, glycocalyx expression of sLe^X^ on either membrane protein scaffolds (*i.e*., glycoproteins) or membrane lipid scaffolds (*i.e*., glycolipids) is sufficient in itself to mediate potent E-selectin ligand activity, thereby enabling cells in the bloodstream to perform the necessary endothelial “Step 1” adhesive interactions to achieve extravasation.^33^ Among human α(1→3)-FTs, FTVI is the most potent in the creation of sLe^X^, with the next most potent being FTVII. FTIII, FTV, and FTVI can each α(1→3)-fucosylate both LacNAc and sLacNAc, thus these three FTs can create both Le^X^ and sLe^X^, respectively (Table 1, entries b, c). FTIV α(1→3)-fucosylates predominantly LacNAc and it has a very modest capacity to engender sLe^X^, thus it is mainly involved in the creation of Le^X^ (Table 1, entries b, c).^21^

**Table 1:**
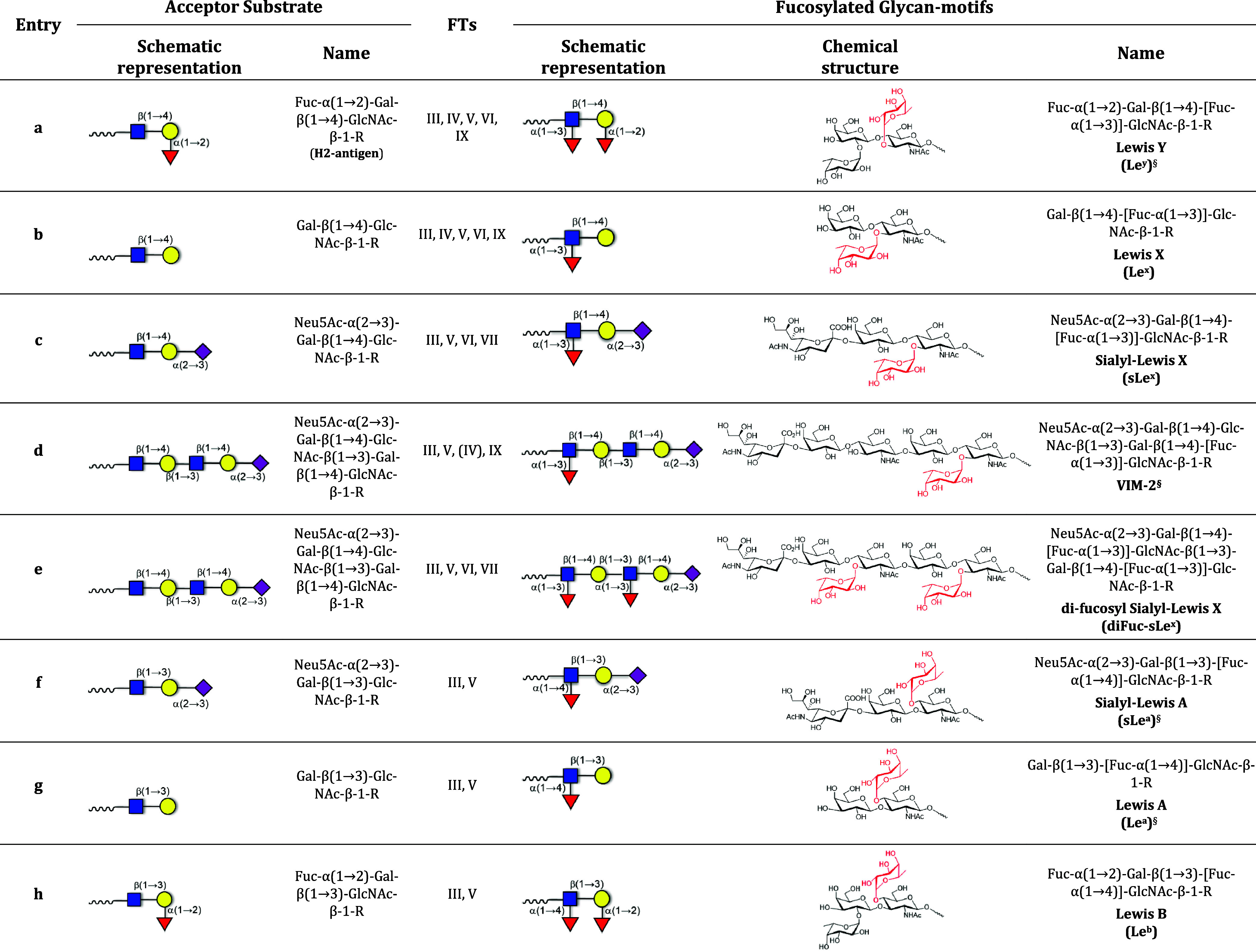
Human FT Acceptor Specificities for the Creation of α­(1→3/4)-Fucosylated Motifs^a^ ^,^ ^21,24^

a ^§^FTs involved in the biosynthesis of the fucosylated motifs are reported in the following references: Le^Y^,^34–37^ Le^a^ and sLe^a^,^38^ and VIM-2.^21,39^

Notably, FTIX can only α(1→3)-fucosylate LacNAc to create the lactosaminyl glycan determinant known as “Lewis X” (Le^X^, CD15; Gal-β(1→4)-[Fuc-α(1→3)]-GlcNAc-β-1-R, Table 1, entry b), while FTVII can only α(1→3)-fucosylate sLacNAc; hence, this FT can only create sLe^X^ (Table 1, entry c). However, within a terminal polylactosaminyl glycan of the glycocalyx, FTIX can fucosylate a penultimate LacNAc unit if the ultimate (*i.e*., nonreducing end LacNAc unit) is either α(2→3)-sialylated (*i.e*., sLacNAc) or α(1→2)-fucosylated (*i.e*., the “H2-antigen”; Fuc-α(1→2)-Gal-β(1→4)-GlcNAc-β-1-R, Table 1, entry a), thereby generating the determinants known as “VIM-2” (CD65s; Neu5Ac-α(2→3)-Gal-β(1→4)-GlcNAc-β(1→3)-Gal-β(1→4)-[Fuc-α(1→3)]-GlcNAc-β-1-R, Table 1, entry d) or Lewis Y (Le^Y^; Fuc-α(1→2)-Gal-β(1→4)-[Fuc-α(1→3)]-GlcNAc-β-1-R, Table 1, entry a), respectively.^37,39^ Moreover, FTIII, FTIV, FTV, and, also FTVI are involved in the biosynthesis of Le^Y^ (Table 1, entry a);^34–36^ notably, the creation of Le^Y^ by α(1→3)-FTs requires the prior synthesis of the (acceptor) H2-antigen, which is controlled by the action of two α(1→2)-FTs (FTI and FTII) that attach a fucose in α(1→2)-linkage on the Gal residue of terminal LacNAc (Table 1, entry a). Moreover, a recent study showed^21^ that FTIII and FTV, with variable capacity of FTIV (Table 1, entry d), can direct the synthesis, in human mesenchymal stem cells, of VIM-2. The same investigators also reported that “difucosyl sLe^X^” (Neu5Ac-α(2→3)-Gal-β(1→4)-[Fuc-α(1→3)]-GlcNAc-β(1→3)-Gal-β(1→4)-[Fuc-α(1→3)]-GlcNAc-β-1-R, Table 1, entry e) is produced by FTIII, FTV, FTVI, and FTVII.

FTIII and FTV are unique in that they exhibit α(1→4)-FT activity in addition to α(1→3)-FT activity, so they can each make also the α(1→4)-fucosylated “Type-1” isomers known as “Lewis A” (Le^a^, Gal-β(1→3)-[Fuc-α(1→4)]-GlcNAc-β-1-R, Table 1, entry g), “sialylated Lewis A” (sLe^a^, Neu5Ac-α(2→3)-Gal-β(1→3)-[Fuc-α(1→4)]-GlcNAc-β-1-R, Table 1, entry f), and “Lewis b” (Le^b^, Fuc-α(1→2)-Gal-β(1→3)-[Fuc-α(1→4)]-GlcNAc-β-1-R, Table 1, entry h). However, these enzymes are not expressed within human hematopoietic-lineage cells. Importantly, sLe^a^ (also known as the “CA19–9 antigen”) also serves as a potent E-selectin ligand (and, additionally, can bind to P-selectin and L-selectin); this tetrasaccharide is characteristically displayed by gastrointestinal malignancies (*e.g*., pancreatic cancer) and promotes their metastasis.^40^

An important caveat is that only Old-World primates (which include hominids) possess genes encoding functional FTIII, FTV, and FTVI. Importantly, mice possess only two FTs (FTVII and FTIV) capable of creating sLe^X^, and mice lack FTs that generate sLe^a^. Accordingly, one must interpret data regarding FT activities derived from murine studies with caution, as investigations using either mouse cells or mouse models do not mirror the α­(1→3/4)-fucosylation capabilities present in humans.

## Inhibition of the Creation of α(1→3)-Fucosylated Glycan Determinants Using Small-Molecule Inhibitors

To date, compounds reported as inhibitors of fucosylation of the glycocalyx can be classified into two main groups: (i) inhibitors of the biosynthesis of GDP-Fucose (GDP-Fuc) and (ii) molecules that bind and inhibit directly the activity of FTs. A list of the compounds described in this Perspective according to the biochemical assays or the cell models that were tested is included in Table 2 (please note that the numbered compounds in Table 2 are linked to chemical structures presented in Figures 3–8).

Notably, the activity of most of these compounds has only been evaluated in biochemical assays, and therefore, data on the direct effects of inhibition of glycocalyx fucosylation remain elusive.^41–47^ In this Perspective, we will provide a comprehensive overview of compounds, reported to date, that belong to both groups, with a particular focus on the comparison of the respective glycan-editing *vs* the glycan-motif editing activity. As previously described,^33^ the term “glycan-motif editing” will refer to the use of nontoxic GT inhibitors that precisely inhibit pertinent α(1→3)-FTs responsible for the creation of a specific fucosylated glycan determinant without off-target effects on irrelevant FTs. In this way, nontargeted FTs continue to function unhindered, performing their intended glycan modifications with resulting highly stereospecific alterations of glycan motifs. Alternatively, the use of compounds that are not selective results in the inhibition of the creation of multiple Lewis antigens. Moreover, concerning inhibitors of α(1→3)-FTs, we will place a spotlight on their selectivity/affinity *vs* the different α(1→3)-FT enzymes and on the assays (bioanalytical vs cell models, Table 2) employed for their evaluation. If available and relevant for the purpose of this review, details of the mechanism of action will be discussed.

### a Inhibition of Biosynthesis of GDP-Fucose (GDP-Fuc)

In the cytosol, the biosynthesis of GDP-Fuc is regulated *via* two distinct pathways (Figure 2).^17,22^ The *de novo* pathway is the predominant^53^ route to produce GDP-Fuc (>90%)^54^ within the cell and consists of a cascade of enzymes that convert glucose and mannose, taken in through specific transmembrane transporters, into GDP-Fuc. Among these enzymes, GDP-man-4,6-dehydratase (GMDS)^55,56^ and the GDP-keto-6-deoxymannose-3,5-epimerase-4-reductase (FX)^57,58^ play key roles in the regulation of GDP-Fuc biosynthesis. Briefly, in the *de novo* pathway, GDP-Fuc is created utilizing GDP-mannose (GDP-Man), which is then converted to GDP-4-keto-6-deoxy-d-mannose by the GMDS enzyme (Figure 2). In turn, the GDP-4-keto-6-deoxy-d-mannose is then converted to GDP-Fuc by the homodimeric NADP­(H) binding protein FX in a two-step process.^58^

**Figure 2:**
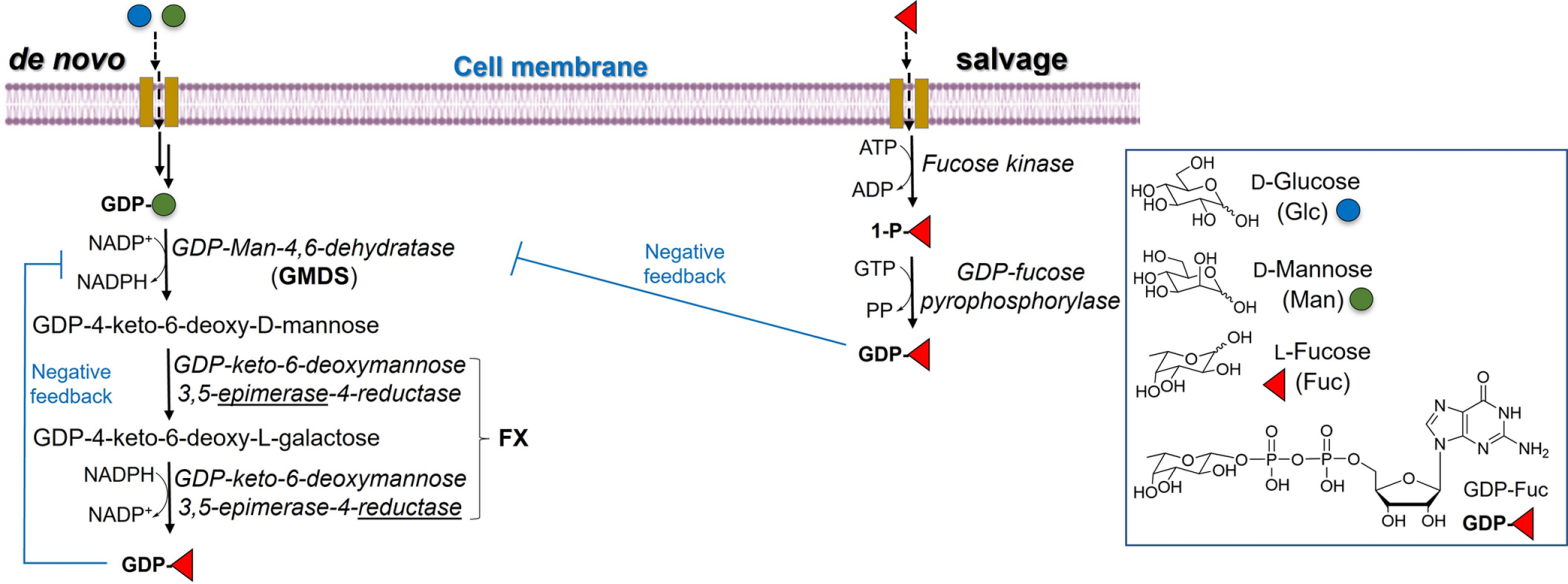
Biosynthesis of GDP-Fuc: steps of the *de novo* pathway *vs* the salvage pathway.^17,20^

The alternative pathway is known as the “salvage pathway”^59^ (Figure 2) whereby fucose, which has been transported into the cytosol from outside the cell or that has been liberated from the catabolism of fucose-containing glycans within lysosomes, is scavenged.^60,61^ The fucose is then transformed into GDP-Fuc by the action of two enzymes: fucose kinase and GDP-fucose pyrophosphorylase.^17,59,62,63^ Whether created *de novo* or *via* alternative pathways, the newly synthesized GDP-Fuc is then shuttled, via a specific transporter (SLC35C1),^64^ into the lumen of the Golgi, where it becomes accessible to the catalytic domain of available FTs. Some experimental evidence showed that a GDP-Fuc transporter is also located in the ER.^65^ −^66,67^ However, despite an ER GDP-Fuc transporter having been identified in *Drosophila*,^66^ the human ER GDP-Fuc transporter remains to be identified. Even though enzymes involved in both the *de novo* or the salvage pathway have been considered prime targets for the discovery of inhibitors of cell surface fucosylation, their targeting results in broad inhibition of the overall fucosylation profile of a cell/protein. Hence, these compounds can be re-evaluated as valuable tools to generally investigate the effects of fucosylation on the biological function of a cell/biomolecule.

Recently, some fucose analogues have been described as GMDS and FX inhibitors. In particular, azido- and alkyne-bearing fucose analogues have been developed as imaging and detection tools for studies focused on the localization, trafficking, and dynamics of glycans.^68^ Thanks to the significant technological advances in the analytical techniques for the study of a cell’s glycoprofile, their mechanism of action has been deciphered. Their cell-permeable per-acetylated derivatives act as metabolic prodrugs and are quickly taken up by cells, converted, inside the cell, into the corresponding GDP-Fuc analogues, and then displayed in the glycocalyx in place of the natural fucose on glycoconjugates.

Only those compounds that result in a clear effect on α(1→3)-fucosylation of the glycocalyx, or in a broad editing of glycocalyx fucosylation (that may also include α(1→3)-fucosylation), will be described in this Perspective.

#### a1 GDP-Keto-6-deoxymannose-3,5-epimerase-4-reductase (FX) Inhibitors

The per-acetylated 6-alkynyl-fucose (**1**, 6-Alk-Fuc, Figure 3) is a pro-drug, and its GDP-Fuc derivative **2** (GDP-6-Alk-Fuc) acts as a competitive inhibitor of the FX enzyme, thus affecting the production of endogenous GDP-Fuc.^54^ In particular, cell lysates were analyzed by LC/MS to assess the amount of GDP-Fuc synthesized and its corresponding alkynylated forms after treatment with **1**. The effect of compound **2** on the FX enzyme was confirmed in both a fluorescence-based assay and in an enzymatic assay using the purified FX.^54^ Compound **1** inhibits the expression levels of a wide range of fucosylated glycans (*e.g*., core fucosylation, Lewis antigens, mono- and difucosylated biantennary and triantennary glycans) in three hepatoma cell lines (*i.e*., Hep3B, HepG2, and FTO2B) and in mammalian cell lines (*i.e*., HEK293 and mouse embryonic fibroblasts). It suppressed cell invasion (but not proliferation), particularly of HepG2 cells, and significantly reduced cell invasion in all three hepatoma cell lines. Notably in a previous report, compound **1** was put forth as an inhibitor of GMDS and FTVIII.^69^ However, Taniguchi and co-workers did not confirm these studies, and they concluded that this compound more potently inhibits FX activity and that inhibition is mediated by direct binding of the corresponding GDP derivative **2** to the catalytic pocket of FX, even when it is used at submillimolar concentration (0.5 mM).^54^

**Figure 3:**
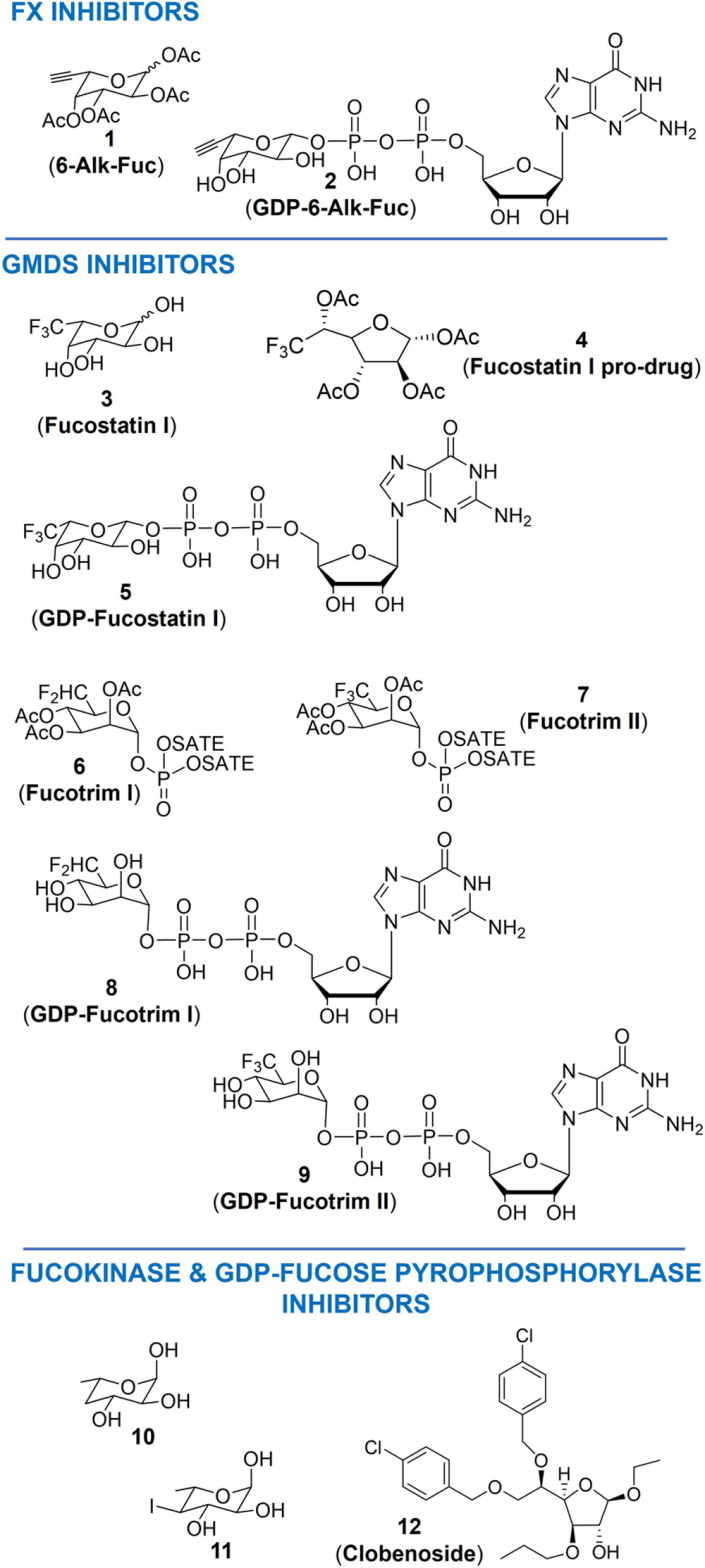
Chemical structures of inhibitors of the *de novo* biosynthesis of GDP-Fuc: GDP-keto-6-deoxymannose-3,5-epimerase-4-reductase (FX) inhibitors (upper panel); GDP-man-4,6-dehydratase (GMDS) inhibitors (middle panel); and fucokinase and GDP-fucose pyrophosphorylase inhibitors (lower panel). SATE: S-acetyl-2-thioethyl.

#### a2 GDP-Man-4,6-dehydratase (GMDS) Inhibitors

Compound **3** (fucostatin I) and its pro-drug, compound **4** (6,6,6-trifluoro-fucose per-*O*-acetate, Figure 3) are metabolically converted into **5** (GDP-Fucostatin I), which acts as an allosteric inhibitor of GMDS. Accordingly, **5** interferes with the *de novo* synthesis of endogenous GDP-Fuc.^70^ The X-ray cocrystal structure of **5** with GMDS was reported and indicated that the compound binds in an allosteric site of the enzyme. Notably, CS-9 CHO cells treated with **4** showed a broad and significant reduction in the expression of cell surface fucosylated glycans (glycocalyx fucosylation was detected using *Lens culinaris* agglutinin (LCA)). However, the effect on α(1→3)-fucosylation of the glycocalyx was not clearly defined.^70^ Then, in 2021, Pijnenborg et al. used the cell-permeable compounds **6** and **7** (fluorinated Fucotrim I and II, Figure 3) as prodrugs. These compounds were metabolically converted into their corresponding GDP derivatives **8** and **9** (GDP-Fucotrim I and II, Figure 3) and successfully decreased both endogenous GDP-Fuc and GDP-Man levels. The activity of these per-acetylated prodrugs was assessed in THP-1, Jurkat, and EL4 cell lines by monitoring fucosylation levels using the fucose-binding lectins *Aleuria aurantia* lectin (AAL) and *Aspergillus oryzae* lectin (AOL).^71^ The mechanism by which compound **8** (GDP-Fucotrim I) acts as a competitive inhibitor of the GMDS enzyme (a recombinantly expressed enzyme was used for this study) was ascertained using NMR spectroscopy by tracking the conversion of GDP-Man into GDP-4-keto-6-deoxymannose at increasing concentrations of **8**.

#### a3 Fucokinase and GDP-Fucose Pyrophosphorylase Inhibitors

A few examples have been reported thus far on small-molecule inhibitors of these enzymes. Reutter and co-workers included in their study several l-fucose analogues, and they found that compounds **10** and **11** ^72^ (Figure 3) are competitive inhibitors (radioligand binding assay using [^14^C]-Fuc, Table 2) of a rat liver fucokinase with *K*_i_ values in the millimolar range (0.5 and 5.0 mM, respectively). Compound **10** significantly affected the uptake of l-fucose into rat hepatoma cells, leading to a decrease of 45% in the incorporation of [^14^C]-Fuc into total cellular glycoproteins.^72^ Moreover, the chloro-containing glucofuranoside **12**, known as **Clobenoside** (Figure 3), impaired both the activity of the fucokinase and GDP-fucose pyrophosphorylase with a *K*_i_ in the range of 5–10 mM.^73^

### b α­(1→3)-FT Inhibitors

Despite the intense efforts to develop novel FT inhibitors, only a few promising approaches are worth mentioning, and most of them provide a gross and FT-family directed glycan editing of the fucosylated glycan determinants. These can be classified into six main classes of compounds according to their structure: (i) donor substrate analogues, (ii) acceptor substrate analogues, (iii) bisubstrate inhibitors, (iv) GDP analogues, (v) glycomimetics, and (vi) nonsugar-related inhibitors (including natural compounds).

#### b1 Donor Substrate Analogues

Structural analogues of the natural donor GDP-Fuc have been used as FT inhibitors, and these can be grouped (Figure 4) into: fluorine-bearing analogues (section 3.2.1.1), GDP-Fuc/fucose analogues (including molecules that contain isostere atoms, moieties that mimic the diphosphate group, and C-6-modified derivatives) (section 3.2.1.2), and transition-state mimics (section 3.2.1.3).

**Figure 4:**
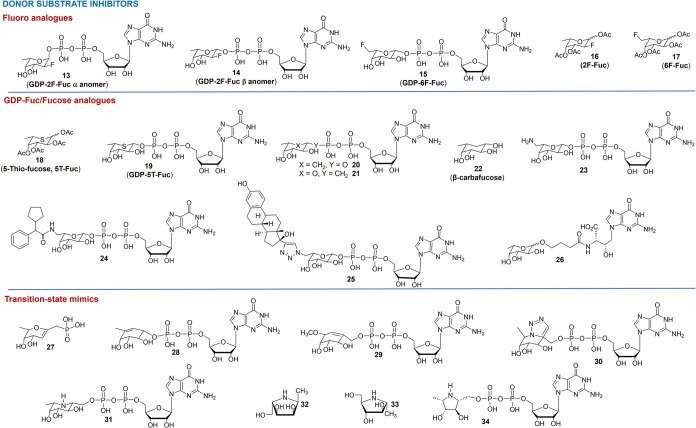
Chemical structures of the donor substrate inhibitors of α(1→3)-FTs described to date: Fluoro analogues (upper panel); GDP-Fuc/fucose analogues (middle panel); and transition-state mimetics (lower panel).

##### b1.1 Fluorine-Bearing Analogues

GDP-fluoro-fucose (GDP-F-Fuc) derivatives **13**–**15** (Figure 4) have been studied in biochemical assays using isolated enzymes (commercially available or cloned) and radiolabeled GDP-[^14^C]-fucose, and they have been proposed as competitive inhibitors of some FTs (*i.e*., FTIII, FTV-VII) with *K*_i_ values in the low micromolar range.^74,75^ In particular, Wong and co-workers described the synthesis of both α- and β-anomers of GDP-2F-Fuc **13** and **14**. Their data demonstrated that the binding sites of FTV and FTVI allowed for stereochemical flexibility. Indeed, despite the unnatural anomeric configuration, α-anomer **13** provided good inhibition of FTs (*K*_*i*_ = 36 μM vs FTV and 2 μM vs FTVI) in the competition radioligand binding assay (Table 2). Conversely, the α-anomer **13** does not inhibit FTIII and FTVII.^75^ Data of the β-anomer **14** on FTV were also confirmed on a complementary assay (Table 2).^74^ The same group also described the chemoenzymatic synthesis of the GDP-6F-Fuc **15**, which had similar *K*_*i*_ values (in the μM range for all the FTs tested, Table 2) compared to **14** (β-anomer GDP-2F-Fuc), as proof that the presence of the fluorine atoms at positions C-2 and C-6 provides similar electronic effects.^75^ The biological activity of these GDP-F-Fuc analogues was further corroborated in cancer cell lines, some years later by Paulson and co-workers.^76^

**Table 2:** Small-Molecule Inhibitors of α(1→3)-Fucosylation, Including Bioanalytical and Cell Assays Used to Test Their Activity and Related References

**INHIBITORS OF THE *DE NOVO* BIOSYNTHESIS OF GDP-FUC (for each number, the pertinent inhibitor structure is provided in Figure 3)**				
**COMPOUND**	**BIOANALYTICAL ASSAY**	**•CELL ASSAY**	***K***_**i**_ **/IC**_**50**_	**REFS**
**TARGET ENZYME: GDP-KETO-6-DEOXYMANNOSE-3,5-EPIMERASE-4-REDUCTASE (FX)**				
**1**	N/A	•mammalian: Mouse embryonic fibroblasts, HEK293 (level of GDP-Fuc and **2**)	N/A	^54^
		•cancer: Hep3B, HepG2, FTO2B (glycocalyx fucosylation)		
**2**	Enzymatic assay and fluorescence-based FX binding assay	•N/A	**2**: *K*_i_ = 2.9 μM (enz. assay)	^54^
**TARGET ENZYME: GDP-MAN-4,6-DEHYDRATASE (GMDS)**				
**4**	N/A	•CS-9 CHO (glycocalyx fucosylation)	N/A	^70^
**5**	Surface plasmon resonance (chip coated with GMDS)	•N/A	**5**: *K*_D_ = 11 μM	^70^
**6–7**	N/A	•THP-1, Jurkat, EL4 (glycocalyx fucosylation)	**6**: IC_50_ = 2.0–38 μM^a^	^71^
			**7**: IC_50_ = 0.45–28 μM^a^	
**8**	Enzyme activity assay (*Readout*: formation of GDP-4-keto-6-deoxymannose by nuclear magnetic resonance (NMR) spectroscopy)	•N/A	**8**: IC_50_ = 231 μM	^71^
**21**	Surface plasmon resonance (chip coated with GMDS)	•N/A	**21**: *K*_D_ = 9 μM	^70^
**TARGET ENZYME: FUCOKINASE & GDP-FUCOSE PYROPHOSPHORYLASE**				
**10**	Radiometric enzymatic assay ([^14^C]-Fuc)	•hepatoma 7777 (glycocalyx fucosylation)	**10**: *K*_i_ = 0.5 mM	^72^
**11**	Radiometric enzymatic assay ([^14^C]-Fuc)	•N/A	**11**: *K*_i_ = 5 mM	^72^
**12**	Radiometric enzymatic assay ([^14^C]-Fuc)	•N/A	**12**: *K*_i_ = 5–10 mM	^73^

**FUCOSYLTRANSFERASE INHIBITORS**				
**COMPOUND**	**BIOANALYTICAL ASSAY**	**•CELL ASSAY**	***K***_**i**_ **/IC**_**50**_	**REFS**
**DONOR SUBSTRATE INHIBITORS (for each number, the pertinent inhibitor structure is provided in Figure 4)**				
*FLUORO ANALOGUES*				
**13**	Radiometric enzymatic assay (*Donor*: GDP-[^14^C]-Fuc; *Acceptor*: LacNAc-β-O-(CH_2_)_5_CO_2_CH_3_; *Readout*: detection of the radiolabeled fucosylated product)	•N/A	**13**: *K*_i_ = 36 ± 4 μM (FTV)	^75^
			**13**: *K*_i_ = 2 ± 1 μM (FTVI)	
**14**	Radiometric enzymatic assay (*Donor*: GDP-[^14^C]-Fuc; *Acceptor*: LacNAc-β-*O*-(CH_2_)_5_CO_2_CH_3_; *Readout*: detection of the radiolabeled fucosylated product)^75^	•N/A	**14**: *K*_i_ = 38 ± 4 μM (FTIII)	^74^,^75^
			**14**: *K*_i_ = 4 ± 0.6 μM (FTV)^b^	
			**14**: *K*_i_ = 10 ± 2.4 μM (FTVI)	
			**14**: *K*_i_ = 21 ± 2 μM (FTVII)	
**15**	Radiometric enzymatic assay (*Donor*: GDP-[^14^C]-Fuc; *Acceptor*: LacNAc-β-*O*-(CH_2_)_5_CO_2_CH_3_; *Readout*: detection of the radiolabeled fucosylated product)^75^	•N/A	**15**: *K*_i_ = 22 ± 10 μM (FTIII)	^75^
			**15**: *K*_i_ = 3.4 ± 1 μM (FTV)	
			**15**: *K*_i_ = 1 ± 0.5 μM (FTVI)	
			**15**: *K*_i_ = 11 ± 2 μM (FTVII)	
**16–17**	N/A	•HL-60, CHO (glycocalyx fucosylation)	N/A	^76^
*GDP-FUC*/*FUCOSE ANALOGUES*				
**18**	N/A	•HL-60, HepG2 (glycocalyx fucosylation)	N/A	^84^
**19**	Enzyme activity assay (*Donor*: GDP-Fuc; *Acceptor*: triantenary, trisialylated *N*-glycan (A3 prozyme); *Readout*: detection of the fucosylated product by capillary electrophoresis coupled with LIF)	•N/A	N/A	^84^
**20**	Radiometric enzymatic assay (*Donor*: GDP-[^14^C]-Fuc; *Acceptor*: Fuc-α(1→2)-Gal-β(1→3)-GalNAc-β(1→3)-Gal-β(1→4)-Glc *vs* α(1→3/4)-FTs from Colo205 cells; *Readout*: detection of the labeled fucosylated product by a liquid scintillator counter)^88^	•N/A	**20**: *K*_i_ = 67.1 ± 9.8 μM (FTV)^86^	^86^,^88^
	Enzyme activity assay (*Donor*: GDP-Fuc; *Acceptor*: LacNAc; *Readout*: fucosylated product formation by electrospray ionization (ESI) mass spectrometry)^86^			
**21**	Enzyme activity assay (*Donor*: GDP-Fuc; *Acceptor*: LacNAc; *Readout*: fucosylated product formation by ESI mass spectrometry)	•N/A	**21**: *K*_i_ = 889 ± 93 μM (FTV)	^86^
**22**	N/A	•HepG2, CHO (glycocalyx fucosylation)	**22**: IC_50_ = 17 ± 8 μM	^83^
**23**	Radiometric enzymatic assay (*Donor*: GDP-[^14^C]-Fuc; *Acceptor*: LacNAc; *Readout*: detection of the radiolabeled fucosylated product by a liquid scintillation counter)	•N/A	**23**: *K*_i_ = 102 ± 17.3 μM and IC_50_ = 104 μM (FTIX)	^89^
**24**	Radiometric enzymatic assay (*Donor*: GDP-[^14^C]-Fuc; *Acceptor*: LacNAc; *Readout*: detection of the radiolabeled fucosylated product by thin-layer chromatography)	•N/A	**24**: *K*_i_ = 0.029 ± 0.009 μM and IC_50_ = 0.094 μM (FTVI)	^90^
			**24**: *K*_i_ = 0.31 ± 0.09 μM and IC_50_ = 0.69 μM (FTV)	
**25**	Enzyme activity assay (*Donor*: GDP-Fuc; *Acceptor*: modified chitotriose; *Readout*: fucosylated product formation by matrix-assisted laser desorption/ionization-Time-of-flight mass spectrometry (MALDI-TOF-MS))	•N/A	**25**: *K*_i_ = 293 nM (FTV)	^91^
				
				
				
*TRANSITION-STATE MIMICS*				
**28**	Enzyme activity assay (*Donor*: GDP-Fuc; *Acceptor*: LacNAc; *Readout*: fucosylated product formation by ESI mass spectrometry)	•N/A	**28**: *K*_i_ = 25.6 ± 2.8 μM (FTV)	^86^
**29**	Enzyme activity assay (*Donor*: GDP-Fuc; *Acceptor*: LacNAc: *Readout*: production of GDP in a fluorimetric assay by combining pyruvate kinase and lactate dehydrogenase)^48^	•N/A	**29**: *K*_i_ = 8 μM (FTV)	^95^
			**29**: *K*_i_ = 6 μM (FTVI)	
**30–31**	Enzyme activity assay (*Donor*: GDP-Fuc; *Acceptor*: LacNAc: *Readout*: production of GDP in a fluorimetric assay by combining pyruvate kinase and lactate dehydrogenase)^48^	•N/A	**30**: *K*_i_ = 8 μM (FTV); *K*_i_ = 13 μM (FTVI)	^95^
			**31**: *K*_i_ = 13 μM (FTV); *K*_i_ = 11 μM (FTVI)	
**32**	Enzyme activity assay (*Donor*: GDP-Fuc; *Acceptor*: NeuAc-α(2→3)-Gal-β(1→4)-GlcNAcβ-*O*-allyl; *Readout*: fucosylated product formation)^49^	•N/A	**32**: IC_50_ = 80 mM (FTV)	^97^,^98^
**33**	Enzyme activity assay (*Donor*: GDP-Fuc; *Acceptor*: NeuAc-α(2→3)-Gal-β(1→4)-GlcNAc; *Readout*: fucosylated product formation)^49^	•N/A	**33**: IC_50_ = 34 mM (FTV)	^97^,^98^
**34**	Enzyme activity assay (*Donor*: GDP-Fuc; *Acceptor*: LacNAc; *Readout*: fucosylated product formation)	•N/A	**34**: IC_50_ = 45 μM and 82 μM (FTV)^c^	^100^
**ACCEPTOR SUBSTRATE INHIBITORS (for each number, the pertinent inhibitor structure is provided in Figure 5)**				
**36**	Radiometric enzymatic assay (*Donor*: GDP-[^14^C]-Fuc; *Acceptor*: LacNAc. *Readout*: detection of the radiolabeled fucosylated product)	•N/A	**36**: *K*_i_ = 0.475 ± 0.096 mM (FTVI)^50^	^102^
**37**	Radiometric enzymatic assay (*Donor*: GDP-[^14^C]-Fuc; *Acceptor*: LacNAc. *Readout*: detection of the radiolabeled fucosylated product)^51^	•N/A	**37**: IC_50_ = 71 μM (FTVI)	^103^
**38**	N/A	•U937 (sLe^X^ editing on the glycocalyx)	N/A	^104^
**39**	Enzyme activity assay (*Donor*: GDP-Fuc; *Acceptor*: LacNAc: *Readout*: production of GDP in a fluorimetric assay by combining pyruvate kinase and lactate dehydrogenase)^48^	•N/A	**39**: *K*_cat_ = 1.6 ± 0.07 [sec^–1^] (FTVI)	^104^
**BISUBSTRATE INHIBITORS (for each number, the pertinent inhibitor structure is provided in Figure 5)**				
**40**	Enzyme activity assay (*Donor*: GDP-Fuc; *Acceptor*: LacNAc; *Readout*: fucosylated product formation)	•N/A	**40**: *K*_i_ = 2.4 mM (FTV)	^105^
**41–42**	Enzyme activity assay (*Donor*: GDP-Fuc; *Acceptor*: [2-(2-pyridylamino)ethyl] β-LacNAc; *Readout*: fucosylated product formation by high-performance liquid chromatography (HPLC) with a fluorescence detector)	•N/A	**41**: *K*_i_ = 41 μM (FTV)	^106^
			**42**: *K*_i_ = 43 μM (FTV)	
**GDP ANALOGUES (for each number, the pertinent inhibitor structure is provided in Figure 6)**				
**43**	Enzyme activity assay (*Donor*: GDP-Fuc; *Acceptor*: LacNAc: *Readout*: production of GDP in a fluorimetric assay by combining pyruvate kinase and lactate dehydrogenase)	•N/A	**43**: *K*_i_ = 270 ± 30 nM (FTV),	^109^,^110^
			**43**: *K*_i_ = 62 ± 3 nM (FTVI)	
**GLYCOMIMETICS (for each number, the pertinent inhibitor structure is provided in Figure 7)**				
**44**	Cell-based assay (exofucosylation of the glycocalyx)	•RPMI-8402, MSC (glycoengineering of the glycocalyx for sLe^X^ and Le^X^ in exofucosylation)	**44**: IC_50_ = 0.72 ± 0.05 mM (FTVII)	^112^
			**44**: IC_50_ = 1.18 ± 0.12 mM (FTVI, sLe^X^)	
			**44**: IC_50_ = 1.11 ± 0.05 mM (FTVI, Le^X^)	
**NONSUGAR DERIVATIVES (for each number, the pertinent inhibitor structure is provided in Figure 8)**				
**45–49**	Enzyme activity assay (*Donor*: fluorescently labeled GDP-Fuc; *Acceptor*: Fetuin; *Readout*: detection of fucosylated fetuin in a fluorescence polarization assay)	•N/A	**45**: IC_50_ = 5.1 μM (FTVI); IC_50_ = >500 μM (FTVII)	^114^
			**46**: IC_50_ = 4.3 μM (FTVI); IC_50_= >500 μM (FTVII)	
			**47**: IC_50_ = 5.3 μM (FTVI); IC_50_ = >500 μM (FTVII)	
			**48**: IC_50_ = 1.8 μM (FTVI); IC_50_ = >500 μM (FTVII)	
			**49**: IC_50_ = 2.1 μM (FTVI); IC_50_ = >500 μM (FTVII)	
**NATURAL COMPOUNDS (for each number, the pertinent inhibitor structure is provided in Figure 8)**				
**50**	Patient-derived organoids (PDOs) detecting cell viability	•COLO205 KATOIII (sLe^X^ editing within the glycocalyx)	**50**: IC_50_ = 140 nM (normal derived organoids)	^116^
			**50**: IC_50_ = 50 nM (gastric cancer-derived organoids)	
**51–52**	Enzyme activity assay (*Donor*: GDP-Fuc; *Acceptor*: pyridylaminated α(2→3)-sialyl lacto-*N*-neotetraose; *Readout*: detection of the fucosylated product by HPLC with a fluorescence detector)	•U937(sLe^X^ and E-selectin ligands, recognized by KM93 mAb, editing of the glycocalyx)	**51**: IC_50_ = 4.8 μg/mL (FTVII); IC_50_ = 28.7 μg/mL (FTVI)	^117^
			**52**: IC_50_ = 5.3 μg/mL (FTVII); IC_50_ = 30.1 μg/mL (FTVI)	
**53–54**	Enzyme activity assay (*Donor*: GDP-Fuc; *Acceptor*: pyridylaminated α(2→3)-sialyl lacto-*N*-neotetraose; *Readout*: detection of the fucosylated product by HPLC with a fluorescence detector)	•N/A	**53**: IC_50_ = 3.9 μg/mL (FTVII)	^118^
			**54**: IC_50_ = 2.4 μg/mL (FTVII)	
**55–57**	Radiometric enzymatic assay (*Donor*: GDP-[^3^H]-Fuc; *Acceptor*: Fetuin; *Readout*: detection of the radiolabeled fucosylated product using a scintillation plate reader)	•N/A	**55**: IC_50_ = 0.06 ± 0.01 μM (FTVII)	^119^
			**56**: IC_50_ = 1.2 μM (FTVII)	
			**57**: IC_50_ = 0.7 ± 0.2 μM (FTVII)	
**58**	Enzyme activity assay (*Donor*: GDP-Fuc; *Acceptor*: coumarin labeled LacNAc; *Readout*: detection of the fucosylated product)^52^	•N/A	**58**: IC_50_ = 11.3 μg/mL (FTV)	^120^
			**58**: IC_50_ = 20.9 μg/mL (FTVI)	

a The IC_50_ values were defined as the concentration where a 50% decrease in lectin (AAL, AOL) binding compared to control was observed.

b *K*_i_ was also determined in a fluorometric assay (*K*_i_: 4.2 ± 0.6 μM),^74^ *i.e*., production of GDP by combining pyruvate kinase (PK) and lactate dehydrogenase.

c The two IC_50_ values are related to 1 and 10 mM of LacNac, respectively.^100^

In particular, they reported an optimized protocol for the synthesis of the per-acetylated fucose analogues **16** and **17** (Figure 4), and they observed that these compounds act as prodrugs and are readily taken up by cells (HL-60 and CHO cells) and converted into the corresponding donor substrate analogs **14** (GDP-2F-Fuc) and **15** (GDP-6F-Fuc), respectively. This is in line with the known promiscuity of the enzymes involved in the GDP-Fuc salvage pathway, which easily accommodate monosaccharides with artificial substituents.^77^ Evidence of this enzyme accommodation clearly emerges in the ability of compounds **16** and, to a lesser extent, **17** to efficiently reduce the expression of both sLe^X^ and Le^X^ in human HL-60 cells by the inhibition of FTIV and FTVII, as well as to block *core* fucosylation of *N*-glycans by the inhibition of FTVIII in CHO cells (Table 2).

Accordingly, this approach overcomes the limitation of GDP-2F-Fuc, which was ineffective in crossing the cell membrane due to the negative charge of the phosphate group. In addition, compound **16** turned out to work not only as a competitive inhibitor of FTs but also as a global metabolic inhibitor. Indeed, the structural similarity of its corresponding nucleotide donor **14** with the natural donor GDP-Fuc permitted its accumulation due to the lack of turnover, and, in turn, shut down the *de novo* synthesis of GDP-Fuc due to existing metabolic feedback loops within these pathways.^76,78^

This study prompted further investigation into the use of **16** as a new therapeutic molecule in cancer settings.^69,79,80^ In 2016, compound **16** was employed in Phase I clinical trials for the treatment of advanced solid tumors, but the clinical trials were eventually terminated due to associated thromboembolic events.^81^ As a consequence of the complete depletion of the natural GDP-Fuc, and the subsequent accumulation of either **14** or **15**, at certain concentrations, the incorporation of these unnatural fucose derivatives into some fucosylated glycans occurred (*i.e*., core fucosylation for **14** and **15** and sLe^X^ for **15**).^76^ The biological effects related to the incorporation of unnatural monosaccharides into glycans have not yet been elucidated. However, the use of this approach for therapeutic interventions raises concerns.^82,83^ On the other hand, compound **16** provides a gross disruption of multiple types of fucosylated motifs, and it was mainly used to study the biological functions of fucosylated glycans in diverse diseases, including cancer.

##### b1.2 GDP-Fuc/Fucose Analogues

Vocadlo and co-workers^84^ studied the inhibition of Le^X^ and sLe^X^ creation by FTIII and FTVII in HL-60 and HepG2 cancer cells using the previously reported compound **18** (5-thio-fucose, 5T-Fuc, Figure 4 and Table 2).^85^ This compound is taken up by cancer cells, and it is metabolically converted into the corresponding nucleotide derivative **19** (GDP-5T-Fuc). Then, compound **19** competes with the endogenous GDP-Fuc, inhibits the expression of fucosylated *N*-glycans produced by FTIII and FTVII, and provides feedback inhibition of GDP-Fuc biosynthesis. Notably, the *core* fucosylation was not affected by treatment with **18**. Moreover, compound **18** is not transferred within the glycocalyx of cancer cells to form sulfur analogs of fucosylated glycans. An enzyme activity assay (Table 2), using commercially available human FTVII and compound **19**, further confirmed that the enzyme does not transfer the thio-fucose on diverse *N*-glycan acceptor substrates.

Carba- and *C*-glycosidic analogs of GDP-Fuc have been developed in an attempt to improve metabolic stability.^70,86–88^ The isosteric analogues **20** and **21** (Figure 4) retained their micromolar inhibitory ability *vs* the commercially available FTV using LacNAc as an acceptor substrate in a mass spectrometry-based assay (Table 2). Moreover, carba-derivative **20** inhibited the activity of α­(1→3/4)-FTs extracted from human colon adenocarcinoma Colo205 cells, in a competition assay using the GDP-[^14^C]-Fuc as the donor and the lacto-*N*-fucopentaose (LNF 1: Fuc-α(1→2)-Gal-β(1→3)-GlcNAc-β(1→3)-Gal-β(1→4)-Glc) as the acceptor substrate. Then, the *C*-glycosidic derivative **21** was also characterized as a competitive inhibitor of the GMDS enzyme using surface plasmon resonance (Table 2).^70^

Recently, the β-carbafucose **22** has been reported as an efficient metabolic inhibitor of fucosylation.^83^ It induces, in human cell lines HepG2 and HL-60, a promiscuous inhibition of fucosylation including α(1→3)-fucosylation. This compound was investigated mainly as a tool to produce afucosylated mAbs.

The synthesis of **23** (GDP-6-amino-β-l-fucose, Figure 4), bearing a terminal amine group at position C-6 of the fucose, has also been recently described.^89^ Kinetic studies using the radioisotope-labeled GDP-[^14^C]-Fuc as a donor and human FTIX revealed that it is a weak inhibitor (*K*_*i*_ = 102 μM, a reduction of less than 25% of the enzyme activity was observed, Table 2). A chemoenzymatic synthesis of **23** was also reported by Lin and co-workers.^90^ Similarly, compound **23** was used as the core component and was coupled to various carboxylic acids, thus resulting in a cohort of GDP-Fuc analogues bearing structurally different hydrophobic moieties at position C-6 of the fucose residue. All these compounds were screened against human FTV, FTVI, and *Helicobacter pylori* α(1→3)-FT, and, among them, compound **24** (Figure 4) resulted in a *K*_i_ of 29 nM *vs* FTVI and 310 nM *vs* FTV (Table 2).^90^

Structural modifications at the C-6 position have been further investigated by Nishimura and co-workers.^91^ Specifically, they synthesized a family of GDP-Fuc analogues by Cu­(I)-catalyzed azide-alkyne cycloaddition (CuAAC) between azido GDP-Fuc derivatives with various alkynes. In doing so, the authors developed a new methodology for the identification of GT inhibitors that utilizes high-throughput quantitative MALDI-TOF MS-based screening. Conjugate **25** (Figure 4) was identified as a competitive inhibitor of recombinant human FTV (*K*_*i*_ = 293 nM, Table 2). Notably, it is not used by FTV as a substrate. A few examples where the diphosphate group of the GDP-Fuc was replaced by nonionic and bioisoster groups have been reported. These compounds have been described as poor inhibitors, with inhibition only evident against FTVIII.^92^

The chemoenzymatic synthesis of the α and β anomers of **26** (Figure 4) has been reported.^93^ The carboxylic group was proposed to mimic the phosphate group in the structure of GDP-Fuc, whereas the hydroxyl group at the β-position of the α-amino acid moiety was expected to behave as one of the hydroxyl groups in position C-2 or C-3 of the ribose. Unfortunately, this approach was unsuccessful, as neither the α or β anomers of **26** showed inhibitory activity toward either FTIII or FTVI.

##### b1.3 Transition-State Mimics

The design of molecules that mimic the transition state (TS) of GTs is one of the earliest approaches used to prepare effective GT inhibitors. These compounds resemble the TS of the enzyme-catalyzed reaction by incorporating either the geometry or the charge of the TS. A few details about the activity of these inhibitors *vs* α(1→3)-FT enzymes have been reported, and data are mainly based on the evaluation of *K*_i_ in bioanalytical assays *vs* some selected FTs.

Schmidt and coauthors reported the synthesis of a phosphonate derivative **27** (Figure 4).^94^ However, to date, details of the biological activity of this compound are not available. Then, Toyokuni and co-workers reported on the synthesis of an unsaturated carbafucose analogue **28** (Figure 4) and observed, for this compound, a *K*_*i*_ = 25.6 μM *vs* FTV. This value is lower than the *K*_i_ (67.1 μM) of its carba analogue **20** (Figure 4).^86^ The preference toward the half-chair conformation of **28** over the stable ^1^C_4_ conformation of **20** is in line with the hypothesis that enzymes bind tightly with their transition states than with substrates.^44^ The flattened half-chair conformation of the fucose moiety in the TS was also included with the GDP-fucose derivative **29** (Figure 4).^95,96^ The same authors also reported two aza-analogues, **30** and **31**. Compound **30** contains a fused triazole ring that mimics the conformation of the fucose in its TS, whereas compound **31** replaces fucose with an aminocyclitol ring that mimics, at physiological pH, the partial positive charge in the TS. All the transition-state mimics **29**–**31** (Figure 4) have an extra methylene group that features an elongated glycosidic bond. A fluorescence-based assay revealed that all of them are competitive inhibitors of FTV and FTVI (*K*_*i*_ = 6–13 μM, Table 2).

The hypothesis that azasugars, previously developed as glycosidase inhibitors, may also work as inhibitors of GTs was anticipated by Wong and co-workers.^97,98^ More recently, an extensive review on the synthesis of iminosugars and efforts for their repurposing as GT inhibitors has been reported.^99^ Wong and co-workers reported on the synthesis of a series of five-member azasugars, and among them, compounds **32** and **33** (Figure 4) proved to be poor inhibitors of α(1→3)-FTs with IC_50_ in a millimolar range (80 mM for **32**, and 34 mM for **33**).^97,98^ Their inhibitory activity increased to the micromolar range when GDP was added in the biochemical assay. The authors speculated that the synergistic effect might be due to an interaction between GDP and the azasugars in the active site of the enzyme, whereby they could form a complex.

Finally, the GDP derivative **34** (Figure 4), bearing a five-membered azasugar instead of the fucose moiety, was reported by Blechert and co-workers as a micromolar inhibitor of FTV in an enzyme activity assay (Table 2).^99,100^

#### b2 Acceptor Substrate Analogues

A few examples of acceptor substrate analogues have been reported to date, and their potency is generally low, with a *K*_i_ in the millimolar range. The first report on acceptor substrates as FT inhibitors was in 1991, when Palcic et al. reported the synthesis of some deoxy analogues of the “Type-1” acceptor substrate.^101^ In particular, they tested the disaccharide **35** (Figure 5) in a competitive assay with the natural acceptor substrate vs α(1→3)-FTs from human serum. No inhibition was observed.

**Figure 5:**
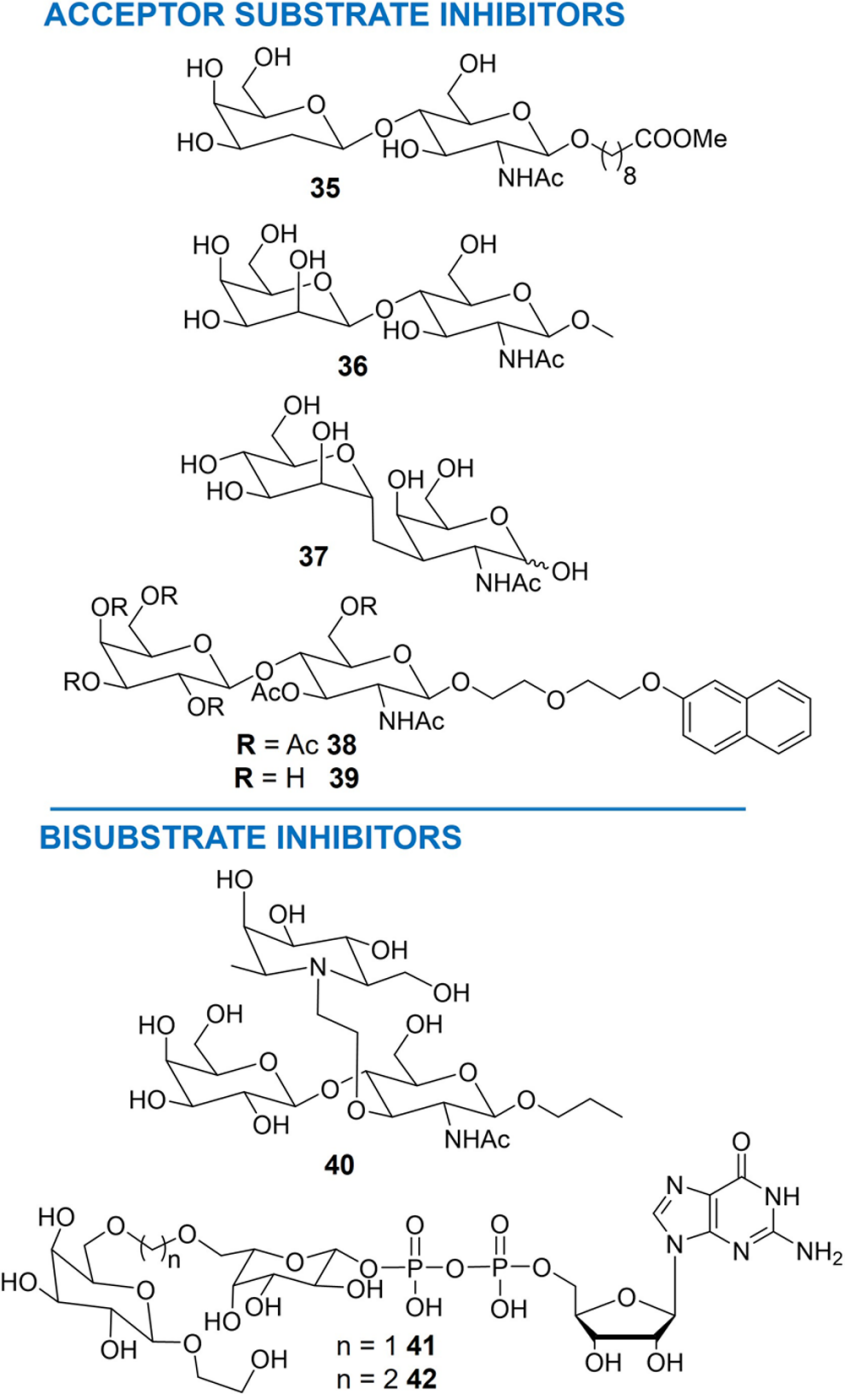
Chemical structures of the acceptor substrate (upper panel) and bisubstrate (lower panel) inhibitors of α(1→3)-FTs described to date.

Then, the epimerization of the hydroxyl group at the C-2 of the galactose residue of LacNAc resulted in the creation of the disaccharide **36** (Figure 5).^102^ This modification resulted in a specific inhibition of FTVI (FTIII, FTIV, and FTV were also included in this study) in a low millimolar range using a radiometric enzymatic assay (Table 2). Normally, the acceptor binds exclusively to the GDP-fucose–enzyme complex. However, the data demonstrated that compound **36** can bind to the GDP-fucose complex, as well as to the native enzyme. Furthermore, the authors observed that compound **36** possibly binds FTVI in a pose different from that of the natural acceptor substrate LacNAc.

Vogel and co-workers reported on the synthesis of the *C*-disaccharide α-d-Man*p*(1→3)­CH_2_–d-GalNAc **37** (Figure 5).^103^ It was tested in a radiometric enzymatic assay (Table 2) using FTVI expressed in *Pichia pastoris*, and it inhibited the enzyme with an IC_50_ of 71 μM. The data demonstrated that it competes with the GDP, suggesting that it is a mimic of the α-l-Fuc­(1→3)-d-GalNAc portion of Le^X^ with the d-mannose residue replacing the fucose residue.

In another attempt at substrate analogs, Wong et al. designed a series of per-acetylated LacNAc decoy acceptors.^104^ They contained different aglycons at the anomeric position of the GlcNAc residue, and their ability to inhibit the expression of sLe^X^ in U937 cells expressing FTIV and FTVII was studied. They reported that the hydrophobicity of the aglycon and the length of the linker play a pivotal role in the inhibitory activity. In particular, per-acetylated compound **38** (Figure 5), which contains naphthyl moieties, provided better inhibition of cell surface expression of sLe^X^ (Table 2). Moreover, kinetic constants for the deprotected derivative **39** were evaluated in an enzymatic assay (Table 2) using as readout the amount of GDP released in the fucosylation reaction of FTVI with GDP-Fuc and LacNAc as the acceptor substrate.

As with most of the donor substrate analogues, the acceptor substrate analogues can be included in the family of FT inhibitors that provide broad glycan editing of the glycocalyx. Their development and use to investigate the biological effect of inhibition of cell surface fucosylation are less explored compared to the donor substrate compounds; however, they represent important tools to explore the space around the acceptor binding site of these enzymes.

#### b3 Bisubstrate Inhibitors

Bisubstrate inhibitors contain the structural features of both the natural donor and acceptor.^105^ In 1996, Wong and co-workers described the synthesis of the azatrisaccharide **40** (Figure 5) in an attempt to create a mimetic Le^X^.^105^ It contains a β-l-homofuconojirimycin residue covalently linked *via* an ethylene spacer to the C-3 position of the GlcNAc residue of the acceptor substrate. The ethylene spacer was used to mimic the partially forming glycosidic linkage; however, computational modeling demonstrated that it is too short, and compound **40** did not result in an ideal mimetic of Le^X^. Instead, it turned out to be a competitive, with respect to the donor GDP-Fuc, inhibitor (*K*_*i*_ = 2.4 mM) of human FTV. Of note, a synergistic effect of **40** with GDP was observed in a biochemical assay on an isolated enzyme with a 77-fold enhancement in its inhibitory potency. These findings confirmed the synergy of azasugars in combination with GDP in the inhibition of FTs.

To overcome limits related to the lack of X-ray crystallographic structures for most of the FTs, the bisubstrate analogues **41** and **42** were designed relying only on the three-dimensional structure of Le^X^.^106^

Notably, position C-6 of the two monosaccharide residues Fuc and Gal was tethered using an alkyl linker (*n* = 1, 2). Then, the LacNAc was replaced with the 2-hydroxyethyl β-d-galactoside to simplify the synthesis, and a 2-hydroxyethyl aglycon was included at the anomeric position of the galactose residue to resemble the hydroxyl group at position C-3 of LacNAc, which is known to be relevant for binding to the enzyme. Compounds **41** and **42** proved to be moderate inhibitors of FTV and FTVI in an enzyme activity competition assay (Table 2). Then, the ability of these compounds to be recognized as substrates by the two selected FTs was also evaluated by monitoring by LC-MS the formation of the product that could result from the transfer of the 6-modified l-Gal moiety to LacNAc. It resulted in compounds **41** and **42** being transferred to LacNAc by FTVI, whereas they were not substrates for FTV.

Some other relevant synthetic efforts to design and prepare bi- and trisubstrate analogues have been reported to date;^107,108^ however, inhibition data have not been included. This area of research would significantly benefit from the discoveries related to the two previously described groups of FT inhibitors. Indeed, the covalent conjugation of selective/potent donors and acceptor substrate FT inhibitors could result in a promising strategy to provide precise fucosylation-motif editing of the glycocalyx.

#### b4 GDP Analogues

The design of GDP analogues as FT inhibitors was originally described by Wong and co-workers.^109,110^ The rationale behind the design of these inhibitors relies on the hypothesis that the GDP moiety is primarily involved in the binding with the FTs, and on the presence of a hydrophobic pocket adjacent to the binding site of the acceptor. On this basis, they reported a straightforward methodology based on CuAAC that allowed them to easily create a library of GDP-triazole derivatives where the fucose moiety was omitted and the GDP core was tethered *via* a spacer of variable length to different hydrophobic groups. A microplate assay was used to screen the synthesized compounds, and compound **43** (Figure 6) turned into a nanomolar inhibitor of FTVI. In particular, it showed a *K*_*i*_ of 62 nM *vs* FTVI, and it also inhibited FTV (*K*_*i*_ = 270 nM), but notably had no effect on FTIII.

**Figure 6:**
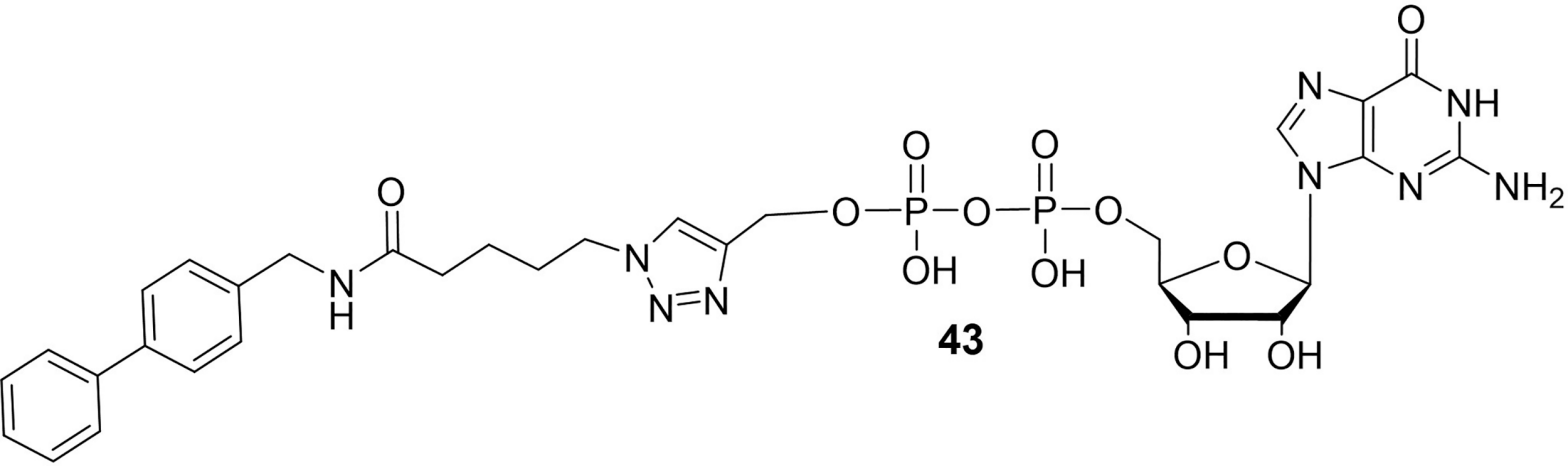
Chemical structure of the GDP analogue **43**.^109,110^

#### b.5 Glycomimetics

Recently, the conformationally constrained fucose mimetic **44** ^111^ (Figure 7) has been repurposed as the next generation of GDP-independent FT inhibitor.^112^ The inhibitory activity of the fucose mimetic was assessed by using an exofucosylation assay on both primary cells and cell lines (Table 2). It is a technique whereby cells that do not natively express either sLe^X^ or Le^X^ determinants but express both the sLacNAc and LacNAc acceptors, respectively, are treated with a pertinent FT together with the donor substrate to stereoselectively install a fucose residue on the acceptor cell surface glycans. Then, the extent of fucosylation in the presence of increasing concentrations of the fucose mimetic is monitored using mAbs that detect the creation of the relevant fucosylated epitopes (CD15s for sLe^X^ and CD15 for Le^X^), while keeping the rest of the cell’s biological functions and its viability intact.

**Figure 7:**
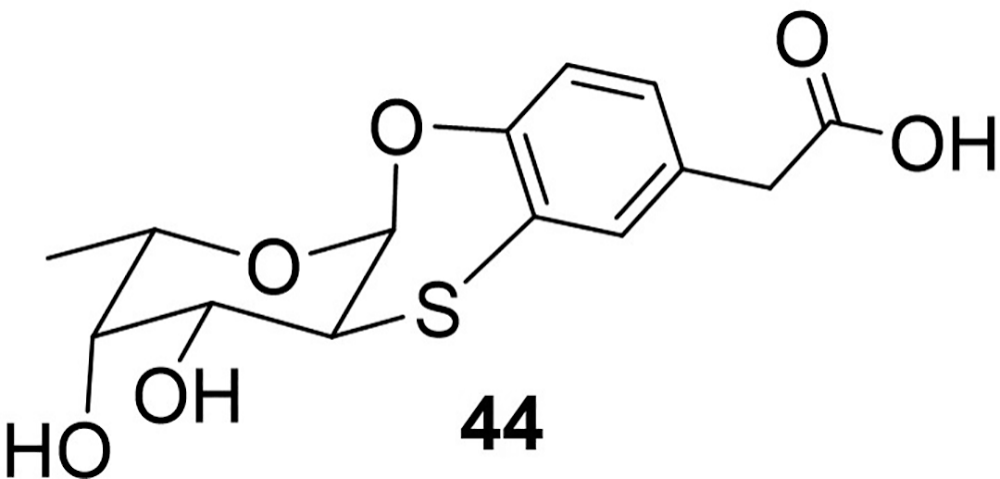
Chemical structure of glycomimetic **44**.^112^

Mimetic **44** selectively and markedly interfered with the activity of FTVI and FTVII in the creation of sLe^x^, but, of note, had no effect on the catalytic activity of FTIX that principally mediates Le^X^ synthesis. This exquisite specificity therefore defines the next generation of selective FT inhibitors, and the structures of **44** and its analogues have been also patented.^113^ Moreover, the use of mimetic **44** affected the ability of the treated cells to adhere to cytokine-stimulated human umbilical vein endothelial (HUVEC) cells under physiologically relevant shear conditions. Interestingly, the fucose mimetic and the natural donor substrate (GDP-Fuc) do not compete for the same enzymatic binding site, highlighting the necessity for further investigations and a deeper understanding of the catalytic activity of these enzymes and the inhibition mediated by this mimetic.

#### b.6 Nonsugar Derivatives

The inhibitory activity of small molecules with variable structures has also been reported.

Paulson and co-workers^114^ developed a fluorescence-based assay for the high-throughput screening of a library of compounds *vs* FTs and sialyltransferases. This assay allowed them to identify several small-molecule inhibitors of FTVI. These compounds contain a 4H-1,2,4-triazole ring (compounds **45**–**47**, Figure 8) or a 4*H*-1,3,4-thiadiazole ring (compounds **48**–**49**, Figure 8) and showed IC_50_ values in the low micromolar range (IC_50_ = 1.8–5.3 μM, Table 2). Furthermore, the activities of some compounds obtained from natural sources have been described. A high-throughput screening (HTS) approach, including a library of 7836 compounds, identified compound **50** ^115^ (Monensin, Figure 8, a polyether antibiotic isolated from *Streptomyces cinnamonensis*) as a promising inhibitor of sLe^X^ expression on cancer cells.^116^

**Figure 8:**
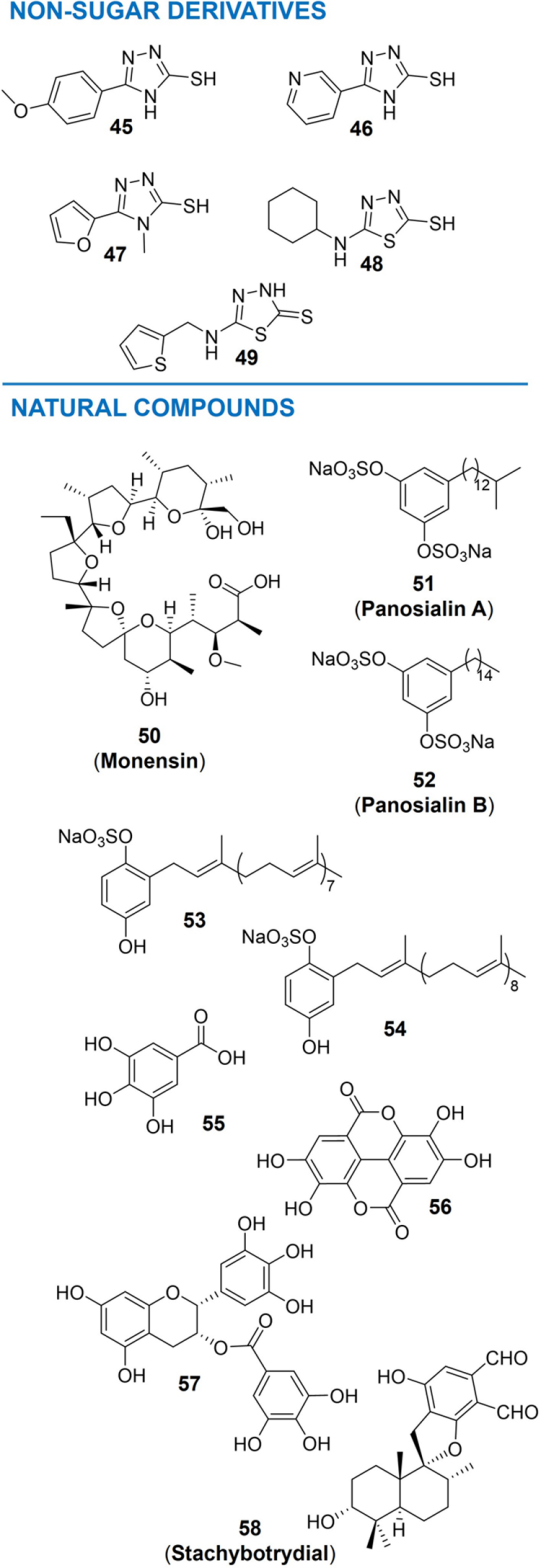
Chemical structure of nonsugar derivatives (upper panel) and natural compounds (lower panel) as inhibitors of α(1→3)-FTs.

The effect of sLe^X^ inhibition was studied *in vitro* on sLe^X^-positive human cancer cell lines (COLO205 and KATOIII, Table 2), indicating that **50** affected protein *O-*glycosylation and secretion, thus resulting in a reduction of cancer cell viability and invasive capacities. In *in vivo* xenograft models of chick embryo chorioallantoic membrane (CAM) and nude mice, **50** reduced tumor formation and invasion.

The benzene disulfonates **51** and **52** (Panosialins A and B)^117^ (Figure 8), isolated from the fermentation broth of *Streptomyces sp*. KY11789, showed an inhibitory profile *vs* FTVII (IC_50_ 4.8 μg/mL for **51** Panosialins A and 5.3 μg/mL for **52** Panosialins B) and FTVI (IC_50_ 28.7 μg/mL for **51** Panosialins A and 30.1 μg/mL for **52** Panosialins B) in an enzyme activity assay (Table 2). Their activity was assessed in both bioanalytical assays using protein A-fused soluble human FTs and in the human promonocytic cell line U937. In particular, they inhibited the expression of sLe^X^, leading to a reduction in adhesion of U937 cells to immobilized E-selectin and to cytokine-activated endothelial cells. The mechanism of inhibition was unclear; however, the authors observed that the activity of both compounds was not affected by the presence of Triton X-100 or Tween-20, thus suggesting that they do not require micelle formation to be active. The polyprenyl-hydroquinone sulfates **53** and **54** (Figure 8), from the lipophilic extract of the Australian marine sponge *Sarcotragus* *sp*., have also been tested and inhibited FTVII with IC_50_ values of 3.9 and 2.4 μg/mL, respectively (Table 2), but had very weak affinity toward FTVI.^118^

Gallic acid **55**, ellagic acid **56**, and (−)-epigallocatechin gallate **57** (Figure 8) are structurally related natural products and are time-dependent and irreversible inhibitors of FTVII.^119^ IC_50s_ of **55**, **56**, and **57** were assessed and resulted in 0.06, 1.2, and 0.7 μM, respectively (Table 2). A series of gallate ester derivatives were also screened, and the data suggested that the gallate (3,4,5-trihydroxybenzoate) structure was the minimum required for FTVII inhibition. However, these compounds are not selective, and they affect the activity of other GTs (*i.e*., FTIV and ST3Gal1) and metalloproteinases (*i.e*., MMP2 and MMP7). Finally, the spirocyclic drimane **58** (named “Stachybotrydial”, Figure 8) was isolated from the culture broth of the fungus *Stachybotrys cylindrospora*.^120^ It is a micromolar inhibitor of FTV and FTVI (Table 2), but its mechanism of action has not been ascertained. Indeed, it does not compete either with the donor GDP-Fuc or with the acceptor LacNAc, and it also inhibits a few sialyltransferases (*i.e*., ST3Gal3, ST3Gal1, and ST6Gal1). Thus, compound **58** does not exclusively inhibit FTs.

## Key Challenges in Drug Discovery for the Inhibition of Cell Surface α(1→3)-Fucosylation

Compared to modulating GT expression *via* genetic composition of the cell, the ability to achieve precise modification of the glycocalyx (glycan-motif editing) using small-molecule inhibitors of GTs holds the advantage of temporally interrupting the expression of a given motif with a much lower risk of adverse effects. Glycoengineering at this level requires the development of nontoxic inhibitors with exquisite specificity for key GTs that regulate the expression of specific disease-associated glycan motifs, ensuring the absence of off-target effects on other enzymes. Despite the relevance of this topic and the huge efforts that have been made toward the development of α(1→3)-FT inhibitors, progress in the field has been significantly hindered by a huge number of unmet needs.

The three-dimensional (3D) structural data of FTIX has been recently reported;^35^ however, no 3D data are available for the other α(1→3)-FTs. A comparison between the X-ray structure of FTIX and the previously reported structure of α(1→3)-FT from the human pathogen *Helicobacter pylori* (HpFucT) has been described. In general, the donor-binding domains of mammalian GT10 FTs show a high degree of sequence similarity and share <21% sequence identity with HpFucT (*i.e*., the GDP-Fuc in HpFucT shows a similar position and interacting residues compared to GDP-Fuc in FTIX).^35^ However, the main differences result in *N*-terminal acceptor domain residues, and these differences eventually drive the acceptor specificity among α(1→3)-FTs. Despite assumptions related to sequence alignments, additional studies are needed to clarify the divergences in the acceptor substrate specificities among the different α(1→3)-FTs. Accordingly, although the X-ray structure of FTIX may help in the creation of homology models for making computational studies, to support the rationale from bioanalytical data, there remains a major bottleneck that limits any notable progress in α(1→3)-FT-targeted drug discovery. Indeed, the large-scale production of α(1→3)-FTs, and, in general of GTs, in amounts required for structural analysis is extremely challenging.^7^ Likewise, this limitation hinders the use of advanced biophysical techniques to unveil enzyme–inhibitor interactions at the molecular level (*e.g*., *via* X-rays and advanced NMR protocols). Therefore, optimized protocols for the expression and purification of such enzymes are highly demanding. For much the same reason, glyco-specific automatized high-throughput screening (HTS) assays, which allow for binding and dose-response measurements of GTs, are poorly explored compared to other targets. Some efforts in this field have been focused on the development of simple and robust HTS methods that avoid the use of radioactivity and make use of minimal amounts of target FTs. This may ensure the transition to large-scale screens of a library of compounds, thus implementing the structural variability (chemical space expansion) in the design of FT inhibitors. Some interesting HTS fluorescence methods have been developed to date;^114^ ,^121^ however, these do not consider the impact of compounds on cells (*i.e*., cytotoxicity, cell uptake, target specificity, subcellular distribution). At present, commercially available kits include non-homogeneous multistep formats or are designed only for screening a few specific enzymes.

As Golgi-resident enzymes, targeting the α(1→3)-FTs poses the challenge of the subcellular delivery of relevant drugs. This point must be taken into account in the design of new inhibitors. No examples of FT inhibitors conjugated to Golgi-targeting moieties have been reported to date, and studies are limited on the development of a few Golgi-targeting probes as fluorescent markers for subcellular labeling.^122,123^

## Concluding Remarks

The glycocalyx comprises an intricate meshwork of oligosaccharide motifs that operationally encodes multiple aspects of cell biology. The use of small-molecule inhibitors able to provide broad and indiscriminate editing of diverse fucosylated glycan motifs within the glycocalyx is an effective approach to study the general role of fucosylation of the glycocalyx in the onset and progression of specific diseases. However, this approach is incapable of modifying the expression of structurally distinct, operationally unique glycocalyx motifs. Accordingly, our aim in creating novel FT inhibitors is at the forefront of efforts to precisely shape cell surface oligosaccharides, a field we call “glycan-motif editing”. Toward this goal, our approach focuses on developing small-molecule inhibitors of key glycosyltransferases that drive the biosynthesis of defined glycocalyx motifs to thereby achieve, with high specificity, an intended physiological effect to improve clinical outcomes for patients in need. This task converges on concepts that exist at the interface of glycochemistry, glycobiology, and clinical medicine, and, as such, the efforts to improve patient outcomes *via* glycan-motif editing serve as a paradigm of the evolving discipline of translational glycobiology.

## Abbreviations

GTs: glycosyltransferases
FTs: fucosyltransferases
GDP: guanosine diphosphate
sLe^X^: sialylated Lewis X
Fuc: l-fucose
Gal: d-galactose
GlcNAc: d-*N*-acetyl-glucosamine
sLe^a^: sialylated Lewis A
Neu5Ac: *N*-acetyl-neuraminic acid
